# Impact assessment of differential chlormequat chloride exposure on soil fungal community dynamics

**DOI:** 10.3389/fmicb.2025.1516835

**Published:** 2025-07-22

**Authors:** Qiujun Lin, Xianxin Wu, Lina Li, Tianshu Peng, Xun Zou, Guang Li, Jianzhong Wang, Chunjing Guo

**Affiliations:** ^1^Institute of Agricultural Quality Standards and Testing Technology, Liaoning Academy of Agricultural Sciences, Shenyang, China; ^2^Agricultural Product Quality and Safety Risk Assessment Laboratory of the Ministry of Agriculture and Rural Affairs, Shenyang, China

**Keywords:** chlormequat chloride, dose-dependent effects, fungal diversity, soil microbial community, peanut cultivation

## Abstract

**Introduction:**

This study investigated the dose-dependent effects of chlormequat chloride (CC) applications on soil fungal community structure and diversity in a peanut cultivation system.

**Methods:**

A controlled field experiment was conducted with four treatment regimes: control (CK, no CC application), low-dose (D, 45g active ingredient/ha), medium-dose (M, 75g a.i./ha), and high-dose (G, 225g a.i./ha). CC solutions were applied during critical growth phases (flowering and pod-setting stages). Rhizosphere soil samples were collected 30-days post-application for microbial analysis. Alpha diversity (e.g., Shannon index), beta diversity (community composition), and functional guild analysis of fungal communities were assessed.

**Results:**

Alpha diversity assessments revealed significant concentration-dependent responses. The low-dose treatment (D) exhibited statistically higher Shannon diversity indices (*p* < 0.05) compared to other treatments. Beta diversity analysis indicated distinct community composition patterns under increasing CC concentrations, characterized notably by a substantial reduction in Ascomycota abundance (from 92.08% in CK to 25.84% in D). Basidiomycota displayed relative stability across treatments. Functional guild analysis identified significant shifts: pathogenic fungi like *Neonectria* spp. declined drastically (from 92.08% in CK to 25.84% under D treatment), whereas saprophytic fungi such as *Plectosphaerella* spp. proliferated markedly (28.68% in D; 22.82% in G vs. 2.26% in CK).

**Discussion:**

These findings establish clear dose–response relationships between CC exposure levels and fungal community parameters. The relative stability of Basidiomycota suggests enhanced tolerance to CC stress compared to Ascomycota. The significant shifts observed in key functional guilds, particularly the decline in pathogens and proliferation of saprophytes, highlight the impact of CC application on soil fungal ecological functions.

## 1 Introduction

Peanuts (*Arachis hypogaea* L.) are a crucial economic and oil crop in China (Zhang and Wang, 2020; Zhou et al., 2022; Zhao et al., 2023; Liu et al., 2022), being the world’s largest producer and exporter (Li et al., 2022). Liaoning is a major peanut growing region in China, with an average annual planting area of approximately 370,000 hectares (Cui et al., 2020). Currently, peanuts rank as the third largest crop in China following corn and rice. Studying fungal diversity in peanut soil is vital for understanding the ecological processes and functions within peanut fields. Soil fungi interact with peanut plants in various ways, such as promoting nutrient uptake, enhancing plant immunity, and regulating plant growth and development (He et al., 2021; Daraz et al., 2024).

Soil, a complex and dynamic ecosystem, harbors diverse microorganisms, including fungi. These soil fungi play vital roles in ecological processes such as nutrient cycling, organic matter decomposition, and soil structure formation (Naitam and Kaushik, 2021; Zhou et al., 2024; Cui et al., 2024). The diversity and composition of the fungal community are critical indicators of soil health and stability, directly or indirectly influencing plant growth, disease resistance, and overall ecosystem functioning (Tong et al., 2021; Fan P. et al., 2022; Fan Z. et al., 2022).

As agricultural modernization advances, the application of plant growth regulators has become increasingly common. Chlormequat chloride (CC) functions by interfering with gibberellin biosynthesis in plants, thereby influencing plant height and morphology (Biswas et al., 2021). It effectively controls excessive plant growth and improves lodging resistance (Lin et al., 2024). However, the specific impacts of CC application on soil fungal communities, particularly in peanut cultivation, remain poorly characterized. Previous studies have demonstrated that agrochemicals can significantly impact soil microbial communities, including fungi (Liao et al., 2021; Hage et al., 2019). These effects can include changes in fungal taxon abundance and composition, as well as alterations in functional activities (Galitskaya et al., 2021; Huang Q. et al., 2021; Huang R. et al., 2021).

Several studies have investigated the effects of plant growth regulators and agrochemicals on soil microbial communities. For example, research on herbicides has shown shifts in fungal community structure and function (Huang Q. et al., 2021; Huang R. et al., 2021; Eichmann et al., 2021). Similarly, pesticide studies have revealed their potential to disrupt fungal diversity and metabolic activities (Zhou et al., 2021). Nevertheless, the specific responses of soil fungal communities to different concentrations of CC in peanut soil require further investigation.

The number of studies investigating chemical impacts on soil ecology is increasing (Li et al., 2024; Han et al., 2023; Bokulich et al., 2018), but further research is needed to explore the effects of CC on soil fungal diversity. This study investigated the effects of different CC concentrations on soil fungal diversity. While enhancing our understanding of the complex relationship between agrochemicals and soil fungi, the study has limitations. Future research should focus on elucidating the specific molecular and biochemical mechanisms underlying fungal responses to CC. Additionally, long-term field trials and multi-site studies are needed to comprehensively understand the cumulative impact of CC on soil fungal diversity and ecosystem services. The results of this study can guide the development of more environmentally friendly and sustainable agricultural practices, aiming to balance crop productivity with the conservation of soil microbial diversity and ecological functions.

## 2 Materials and methods

### 2.1 Field experiments

The study was conducted during the 2023 growing season in Xiaochengzi Town, Kangping County, Shenyang City, Liaoning Province, China (123°35′46″E, 42°75′08”N; Supplementary Figure S1). The experimental site featured a typical temperate continental monsoon climate. A randomized complete block design was implemented with four treatments across 12 plots (5 m × 6 m each), separated by 50 cm buffer zones to prevent cross-contamination.

Commercial 50% chlormequat chloride (CC; Sichuan Runer Technology Co., Ltd., China) was diluted to achieve the following concentrations:

Low-dose (D): 45 g active ingredient (a.i.) ha^−1^.
Medium-dose (M): 75 g a.i. ha^−1^.
High-dose (G): 225 g a.i. ha^−1^.

Foliar applications were conducted on 5 July 2023 during critical reproductive stages of *Arachis hypogaea* cv. baisha, corresponding to flowering (30% open flowers) and initial peg penetration. A calibrated knapsack sprayer was used to uniformly deliver treatments. Applications occurred between 06:00–08:00 under wind velocities <2 m s^−1^ to minimize drift.

### 2.2 Rhizosphere soil sampling

Rhizosphere soil collected on 5 August 2023 (30 days post-treatment), corresponding to the full pod-fill stage. Sampling followed standardized protocols: five representative plants per plot were uprooted using stainless steel trowels sterilized with 75% ethanol. Rhizosphere soil (0–20 cm depth) adhering to roots within 5 mm of the root surface was collected by vigorous shaking. Composite samples were homogenized through a 2 mm mesh to remove coarse debris. Visible roots and stones were manually removed using sterile forceps. Subsamples were flash-frozen in liquid nitrogen within 15 min of collection and stored at −80°C until analysis.

### 2.3 DNA extraction

Genomic DNA was extracted from fresh soil samples (0.5 g aliquots) using the Soil DNA Spin Kit (MP Biomedicals, USA) following the manufacturer’s specifications. The protocol included sequential steps of cell lysis, protein precipitation, and DNA adsorption onto silica membranes. Post-extraction, DNA quality was assessed through:

1. Concentration and purity measurement using a ND 2000 UV–Vis spectrophotometer (Thermo Scientific, USA), recording A_260_/A_280_ and A_260_/A_230_ ratios.
2. Structural integrity verification by 1% agarose gel electrophoresis (100 V, 30 min) with ethidium bromide staining. DNA was stored long-term at −20°C in TE buffer (10 mM Tris–HCl, 1 mM EDTA, pH 8.0).

### 2.4 ITS gene amplification and sequencing

Fungal ITS1 regions were amplified using primer pair F:5’-CTTGTCATTTAGGGAAGTAA-3′ and R:5’-GCTGCGTTCTATCGATGC-3′ in 20 μL reaction volumes containing: 4 μL 5 × Fast Pfu buffer, 2 μL 2.5 mM dNTP mix, 0.8 μL each primer (5 μM), 0.4 μL Fast Pfu DNA polymerase (2.5 U/μL), 10 ng template DNA, and nuclease-free water to volume. Thermocycling parameters (Eppendorf Mastercycler®): initial denaturation at 95°C for 5 min; 27 cycles of denaturation at 95°C for 30 s, annealing at 55°C for 30 s, extension at 72°C for 45 s, final extension at 72°C for 10 min.

Triplicate PCR reactions were pooled. Target amplicons (~300–400 bp) were size-selected by 2% agarose gel electrophoresis, excised, and purified using the AxyPrep DNA Gel Extraction Kit (Axygen Biosciences, USA). DNA concentrations were determined using the QuantiFluor™ ST system (Promega, USA) with dsDNA-specific fluorescence detection.

Purified amplicons were processed by Personalbio (Shanghai, China) for: Illumina-compatible library construction with dual-index barcoding, quality control using an Agilent 2,100 Bioanalyzer, paired-end sequencing (2 × 250 bp) on an Illumina MiSeq platform (Illumina, USA), and demultiplexing to generate FASTQ files.

### 2.5 Sequence processing

Sequence processing was performed using QIIME2 software (v2019.4). Primer sequences were removed using qiime cutadapt trim-paired, discarding sequences with unmatched primers. Quality control, denoising, merging of paired-end reads, and chimera removal were performed using qiime dada2 denoise-paired. These steps were performed per sample library. After denoising all libraries, Amplicon Sequence Variant (ASV) feature sequences and ASV tables were merged, and singleton ASVs (i.e., ASVs with a total count of 1 across all samples) were removed. High-quality sequence length distributions were statistically analyzed using R scripts. To account for uneven sequencing depth, samples were rarefied to 95% of the minimum sample sequence count using QIIME2’s feature-table rarefy function, facilitating robust comparisons of observed ASVs and their relative abundances.

### 2.6 Taxonomic annotation

Taxonomic classification was performed using the QIIME2 classify-sklearn algorithm (Bokulich et al., 2018; https://github.com/QIIME2/q2-feature-classifier) against the UNITE database (v8.3, dynamic version; Koljalg et al., 2013). Default parameters were used.

### 2.7 Co-occurrence network analysis

Co-occurrence networks were constructed based on Spearman correlation coefficients (*ρ* > 0.6 or *ρ* < −0.6) calculated using the R Hmisc package. The top 100 nodes by average abundance were selected to construct the dominant network, visualized using the ggraph package. Negative correlations were excluded to construct a co-occurrence network. The co-occurrence network was modularized using the “multi-level modularity optimization algorithm” from the igraph package. .gml files were generated for visualization in Gephi (Bastian et al., 2009) and Cytoscape (Shannon et al., 2003). The Zi-Pi threshold method was used to classify nodes: Zi > 2.5 and Pi > 0.62 as network hubs; Zi > 2.5 and Pi < 0.62 as module hubs; Zi < 2.5 and Pi > 0.62 as connectors; the remainder as peripheral nodes.

### 2.8 Data statistics

Data were preliminarily organized using Microsoft Office Excel 2021. Alpha diversity indices (Shannon, Simpson, Chao1, Pielou, Observed species) were calculated using QIIME2. Beta diversity was assessed via Principal Coordinates Analysis (PCoA) based on Bray-Curtis distances. One-way ANOVA with LSD post-hoc testing (SPSS 22.0, IBM Corp.) was used for cross-group comparisons of diversity indices and taxonomic abundance differences. Significance threshold: α = 0.05 for all tests.

## 3 Results

### 3.1 Community diversity analysis

#### 3.1.1 Alpha diversity

**Figure 1:**
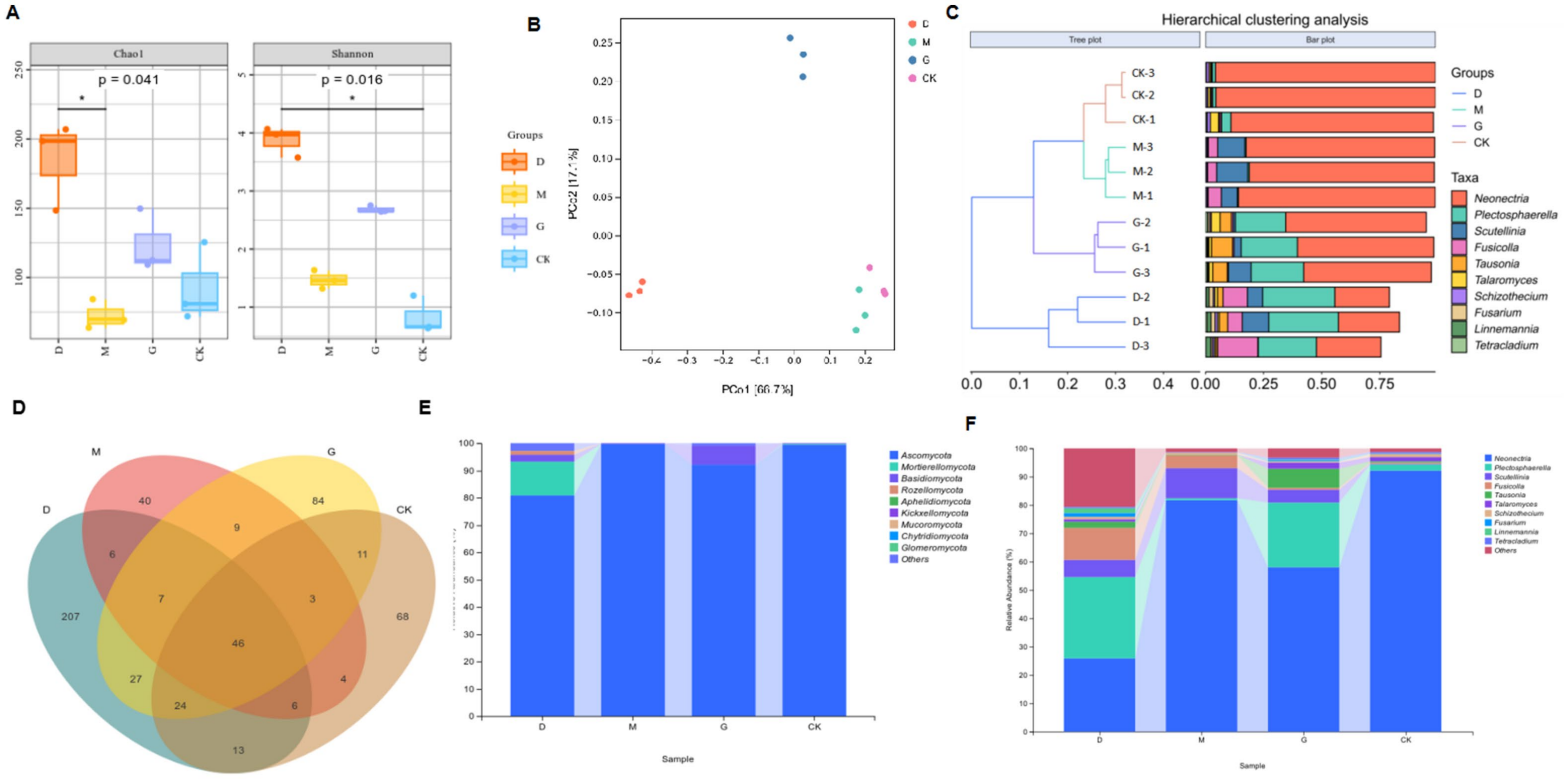
Analyses of microbial communities across different concentrations of CC. **(A)** Chao1 and Shannon diversity indices of the microbial communities in each group, highlighting significant differences (*p*-values indicated). **(B)** Principal Coordinate Analysis (PCoA) plot based on Bray–Curtis dissimilarity, showing the separation of microbial communities among different groups. **(C)** Hierarchical clustering analysis results, with a tree plot depicting sample relationships and a bar plot showing the relative abundances of various taxa. **(D)** Venn diagram illustrating the shared and unique operational taxonomic units (OTUs) among different groups. **(E)** Stacked bar chart presenting the relative abundances of major fungal phyla in each group. **(F)** Stacked bar chart showing the relative abundances of specific microbial genera in each group.

High-throughput sequencing coverage exceeded 99% across all treatments (Table 1), confirming robust sampling depth. Alpha diversity indices (Chao1, Shannon, Simpson, Pielou, Observed species) revealed significant variation among treatments (Figure 1A). The D (low CC) and G (high CC) treatments exhibited higher richness and diversity compared to the M (medium CC) and CK (control) groups, with D showing the highest values. Specifically:

**Table 1:** Alpha diversity index.

Group	Chao1	Goods_coverage	Observed_species	Pielou_e	Shannon	Simpson
D	184.83233a	0.99978	181.50000a	0.51643a	3.87068a	0.85460a
M	72.49420c	0.99988	70.36667c	0.24054c	1.47073c	0.36268c
G	123.93967b	0.99982	120.76667b	0.38946b	2.68390b	0.66973b
CK	92.66967ab	0.99983	89.76667bc	0.12743d	0.83225d	0.17212d

a–d Represent significant differences between groups.

Richness: Chao1 and Observed_species indices ranked as D > G > CK > M (all *p* < 0.05). Diversity: Shannon and Simpson indices followed D > G > M > CK (all *p* < 0.05), while Pielou’s evenness index also showed D > G > M > CK (*p* < 0.05) (Table 2).

**Table 2:** SD of alpha diversity index.

SD	Chao1	Goods_coverage	Observed_species	Pielou_e	Shannon	Simpson
D	31.45274837	4.92375E-05	30.4289336	0.017668493	0.261449447	0.007925618
M	10.61295824	4.83839E-05	11.4718496	0.030010687	0.157693637	0.046442019
G	22.8453802	3.1628E-05	22.8963607	0.02004666	0.057133836	0.030426032
CK	28.63852216	9.17406E-05	26.06076233	0.040020478	0.316756149	0.078316862

#### 3.1.2 Beta diversity

Principal coordinates analysis (PCoA, Figure 1B) based on Bray-Curtis distances explained 83.8% of the variance (PCo1: 66.7%, PCo2: 17.1%). The CK and M groups clustered closely (*p* < 0.05), suggesting minimal structural divergence between these treatments. The D and G treatments formed distinct clusters, significantly differing from each other and from CK and M (Figure 1C). Hierarchical clustering at the OTU level further confirmed these trends, with D and G grouping separately from CK and M (Figure 1C). OTU counts varied markedly: D (336) > G (211) > CK (175) > M (121) (Figure 1D). Only 46 OTUs were shared across all treatments, highlighting CC-driven divergence in fungal community structure.

### 3.2 Taxonomic composition of fungal communities

#### 3.2.1 Phylum-level distribution

Dominant phyla across treatments included Ascomycota (55–92%), Mortierellomycota (3–25%), Basidiomycota (2–15%), Rozellomycota, and Aphelidiomycota (Figure 1E). Ascomycota abundance decreased by 18.73% in D and 7.41% in G compared to M. Mortierellomycota and Basidiomycota exhibited inverse trends, with higher relative abundances in D and G treatments. The D group harbored the highest phylum-level diversity (Table 3).

**Table 3:** Proportion of fungal structures treated at the phylum level.

Phylum	D	M	G	CK
Ascomycota	80.80%	99.64%	92.12%	99.53%
Mortierellomycota	12.48%	0.05%	0.12%	0.07%
Basidiomycota	2.51%	0.30%	6.84%	0.33%
Rozellomycota	1.41%	0.00%	0.06%	0.00%
Aphelidiomycota	0.00%	0.00%	0.00%	0.00%
Kickxellomycota	0.00%	0.00%	0.00%	0.00%
Mucoromycota	0.00%	0.00%	0.00%	0.00%
Chytridiomycota	0.00%	0.00%	0.00%	0.00%
Glomeromycota	0.00%	0.00%	0.00%	0.00%
Others	2.81%	0.01%	0.86%	0.06%

#### 3.2.2 Genus-level dynamics

Top genera included Neonectria (25.8–92.1%), Plectosphaerella (0.6–28.7%), Scutellinia (0.2–10.6%), Fusicolla (0.5–11.3%), and Tausonia (2.4–8.9%) (Figure 1F; Table 4). Neonectria dominated CK soils (92.1%), but its abundance dropped significantly to 25.8% in D. The D treatment enriched Plectosphaerella (28.7%) and Fusicolla (11.3%). The M treatment favored Scutellinia (10.6%). The G treatment elevated Tausonia (8.9%) and Talaromyces (6.2%).

**Table 4:** Proportion of fungal structures treated at the phylum level.

Genus	D	M	G	CK
*Neonectria*	25.84%	81.79%	58.03%	92.08%
*Plectosphaerella*	28.68%	0.58%	22.82%	2.26%
*Scutellinia*	6.04%	10.63%	4.57%	0.22%
*Fusicolla*	11.34%	4.56%	0.62%	0.51%
*Tausonia*	2.24%	0.26%	6.74%	0.26%
*Talaromyces*	0.86%	0.08%	2.17%	1.73%
*Schizothecium*	0.86%	0.58%	0.49%	1.09%
*Fusarium*	1.42%	0.09%	0.53%	0.12%
*Linnemannia*	1.67%	0.01%	0.08%	0.02%
*Tetracladium*	0.42%	0.08%	0.74%	0.36%
Others	20.63%	1.34%	3.20%	1.34%

### 3.3 Multivariate analysis of community structure

#### 3.3.1 Principal component analysis (PCA)

**Figure 2:**
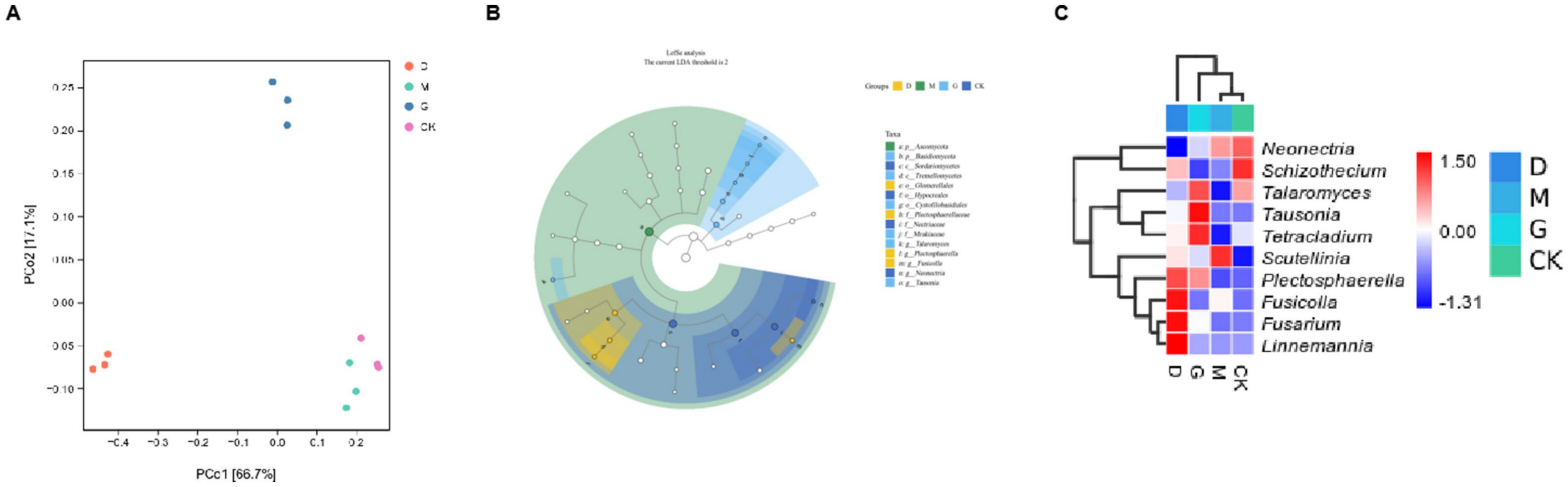
Analyses of microbial communities across different concentrations of CC. **(A)** Principal Coordinate Analysis (PCoA) plot based on microbial community data. **(B)** A circular phylogenetic or compositional analysis graph, likely showing the relationships and relative abundances of different microbial taxa within the groups. **(C)** A heatmap combined with a hierarchical clustering tree.

PCA explained 95% of the variance (PC1: 87.6%, PC2: 7.4%) (Figure 2A). Key findings: CK and M groups clustered tightly, indicating minimal structural shifts at the medium CC dose. D and G treatments diverged significantly, aligning with their distinct alpha and beta diversity profiles.

#### 3.3.2 LEfSe biomarker identification

LEfSe analysis identified 15 biomarkers (LDA score ≥ 4) across taxonomic ranks (Figure 2B). Key biomarkers per group were:

D Group: Enriched in *Fusicolla*, *Fusarium*, *Linnemannia*.
G Group: Dominated by *Tausonia*, *Talaromyces*, *Tetracladium*.
CK Group: Characterized by *Neonectria* (92.1%).
M Group: Characterized by *Scutellinia* (10.6%).

Changes in species composition under different CC concentrations are illustrated in Figure 2C. The most abundant genus in CK was *Neonectria*. Genera more abundant in D included *Fusicolla*, *Fusarium*, *Linnemannia*, and *Plectosphaerella*. The genus more abundant in M was *Scutellinia*. Genera most abundant in G included *Talaromyces*, *Tausonia*, and *Tetracladium*.

### 3.4 Co-occurrence network analysis

#### 3.4.1 Core symbiotic module

**Figure 3:**
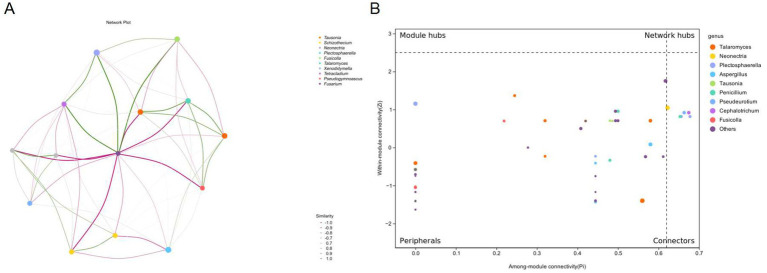
Co-occurrence network and topological roles of fungal genera under different chlormequat chloride treatments. **(A)** Co-occurrence Network Plot: Network visualization depicting significant Spearman correlations (*p* > 0.6 or *p* < −0.6) among dominant fungal genera. Nodes represent genera and edges represent correlation strength, with solid lines indicating positive correlation and dashed lines indicating negative correlation (see legend). Node size is proportional to average relative abundance. Strong negative correlations (all *p* < −0.1, ranging from −1.0 to −0.1) were observed, with values shown in the similarity legend. **(B)** Zi-Pi Plot of Topological Roles: Classification of fungal genera based on network topology parameters. Within-module connectivity (Zi) quantifies a node’s importance within its module; Among-module connectivity (Pi) measures a node’s role in connecting different modules. Nodes are classified into four categories: Network hubs (Zi > 2.5 & Pi > 0.62): Highly connected hubs linking multiple modules. Module hubs (Zi > 2.5 & Pi < 0.62): Hubs central within their own modules. Connectors (Zi < 2.5 & Pi > 0.62): Genera linking multiple modules. Peripherals (Zi < 2.5 & Pi < 0.62): Genera with low connectivity within and between modules. Key genera are labeled by name.

*Talaromyces* exhibited a strong positive correlation with *Fusarium* (*p* ≈ +0.83), suggesting potential functional coupling for synergistic lignocellulose degradation. *Fusicolla* acted as a bridge node, simultaneously linking *Talaromyces* (*p* = +0.79) and *Plectosphaerella* (*p* = +0.68), demonstrating its central role in the carbon source metabolism network. A significant positive correlation (*p* = +0.76) between the cold- tolerant genus *Pseudogymnosus* and *Neonectria* suggested the formation of a low-temperature adaptation alliance. The positive connection (*p* = +0.61) between *Xenodidymilla* and *Tetracladium* implied synergistic potential in organic phosphorus mineralization (Figure.3A).

#### 3.4.2 Negative interaction network

A near-complete negative correlation (p∈[−0.72, −0.89]) existed between *Schizophyllum* and the core symbiotic module (*Talaromyces-Fusarium*), indicating occupation of an independent ecological niche through nutritional competition. *Tausonia* was strongly negatively correlated with *Pseudogymnosus* (*p* = −0.91), reflecting significant differences in nitrogen source utilization strategies. The negative connection (*p* = −0.67) from *Neonectria* to *Xenodidymolla* may characterize competitive exclusion mediated by antibacterial secondary metabolites (Figure 3A).

#### 3.4.3 Topological properties

Network hubs (*Talaromyces*-*Fusarium*) synergistically regulated>65% of community connectivity edges, consistent with a “rich-get-richer” effect. Hub nodes exhibited multifunctional metabolic characteristics. The difference in average connectivity between peripheral node clusters (Pi < 0.1) and hub nodes was 14.7-fold (*p* < 0.001), indicating strong habitat filtering within the community. The Pi values (0.21–0.74) of the connectors (*Pseudogymnoascus*, *Tetracladium*) were significantly higher than those of peripheral nodes (p < 0.001), confirming their role in maintaining network integrity (Figure 3B).

### 3.5 Metabolic pathway modulation by CC treatments

**Figure 4:**
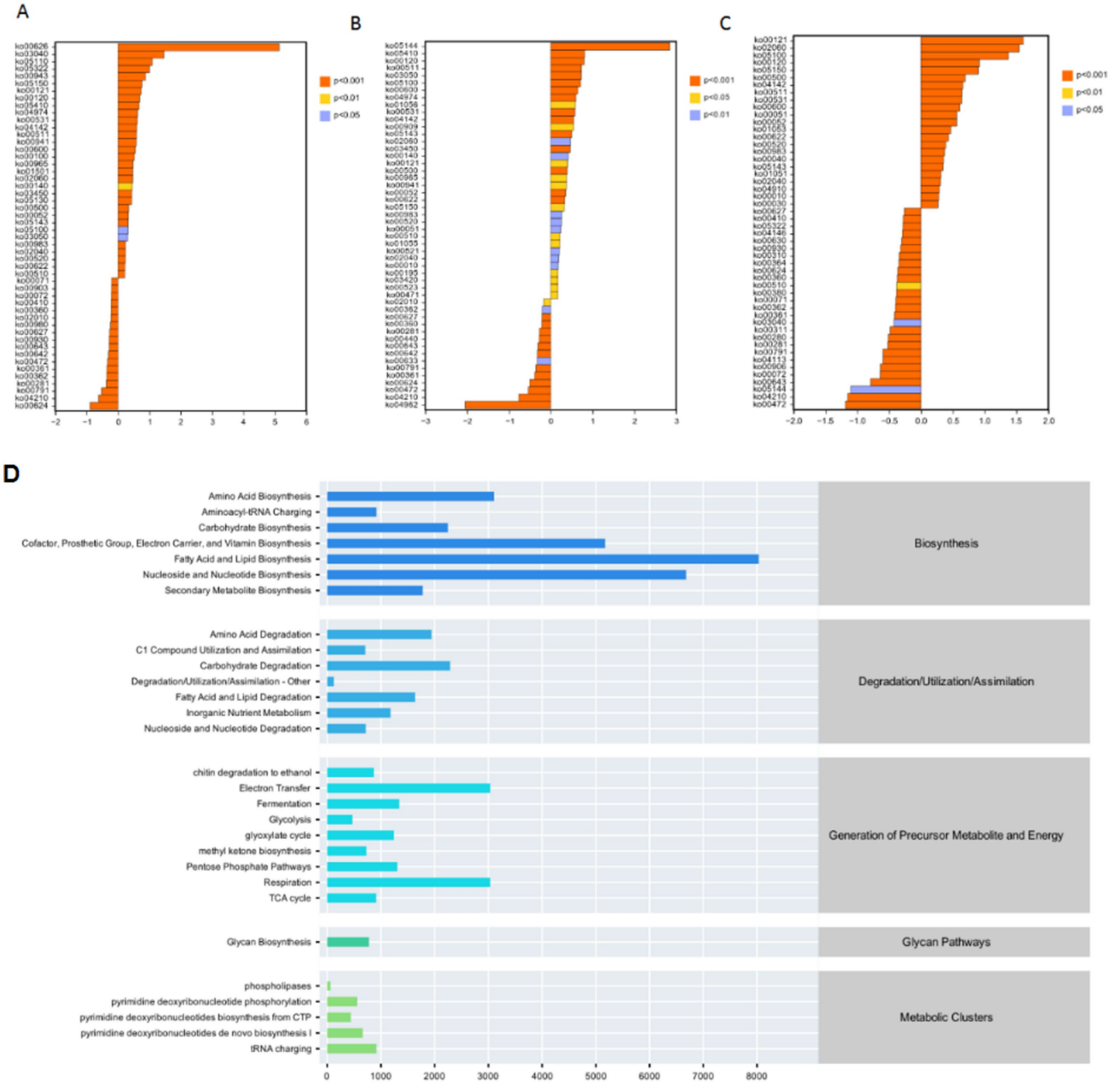
Analyses of metabolic pathways and gene-related distributions in microbial communities. **(A–C)** These panels show histograms representing the distribution of certain gene - related data (possibly gene expression levels or other quantitative metrics), with different colors indicating different significance levels (e.g., *p* < 0.01, *p* < 0.05, *p* > 0.05). The x-axis likely represents the values of the measured variables, while the y-axis shows the number or frequency of data points. **(D)** A bar chart displaying the relative abundances or contributions of various metabolic pathways.

Functional prediction revealed 29 metabolic pathways (Figure 4D), categorized as: Biosynthesis (7 pathways): Fatty acids, nucleotides, cofactors, vitamins. Degradation/Utilization (7 pathways): Carbon, nitrogen, xenobiotics. Energy Production (9 pathways): Glycolysis, TCA cycle, oxidative phosphorylation. Key Regulation (vs. CK, *p* < 0.001): Upregulated: Monoacylglycerol metabolism, NAD/NADP mitochondrial interconversion. Downregulated: Gluconeogenesis, phosphatidylglycerol biosynthesis (Figures 4A–C).

## 4 Discussion

### 4.1 Impact of CC concentration on fungal community diversity

Our results demonstrate that CC concentration is a critical factor determining soil fungal community diversity. Alpha-diversity analysis showed the D (low CC) treatment had the highest richness and diversity indices among all groups. This finding aligns with previous research suggesting low-level applications of certain chemicals can promote the growth and co-existence of diverse fungal species (Fan P. et al., 2022; Fan Z. et al., 2022). The G (high CC) treatment also exhibited increased richness and diversity compared to CK and M, although to a lesser extent than D. This indicates that within a specific concentration range, CC can positively impact fungal community complexity. However, the M group exhibited lower richness than CK (Chao1, Observed_species), despite a slightly higher Shannon index. This complex response may result from the medium CC concentration creating a sub-optimal environment for some species, reducing overall richness while allowing for a more even distribution among the remaining species.

Beta-diversity analysis confirmed CC’s impact on community structure. The distinct separation of D and G treatments from CK and M in the PCoA plot and hierarchical clustering, combined with the small number of shared OTUs (46), clearly shows that CC significantly modifies fungal community composition, particularly at low and high concentrations. Such divergence has implications for soil ecosystem functions, as different fungi play unique roles in nutrient cycling, decomposition (Bardgett and van der putten, 2014; Zhao et al., 2021), and plant-microbe interactions (Nannipieri et al., 2003).

### 4.2 Shifts in fungal taxonomic composition

At the phylum level, CC treatments significantly affected the relative abundances of Ascomycota, Mortierellomycota, and Basidiomycota. The dominance of Ascomycota decreased in D and G treatments compared to M. Conversely, Mortierellomycota and Basidiomycota increased in D and G. Similar shifts have been reported in response to other chemical inputs (Huang Q. et al., 2021; Huang R. et al., 2021), likely reflecting differential tolerance. Ascomycota, being highly diverse, may contain more sensitive species, while Mortierellomycota and Basidiomycota may harbor species better adapted to CC-altered conditions. Basidiomycota’s relative stability further suggests enhanced tolerance.

At the genus level, significant changes occurred in *Neonectria*, *Plectosphaerella*, *Scutellinia*, *Fusicolla*, *Tausonia*, and *Talaromyces*. The drastic decline of the potentially pathogenic or bioactive compound-producing *Neonectria* (Luo and Zhuang, 2010) from 92.08% in CK to 25.84% in D may impact plant health and soil chemical processes. Conversely, D treatment enriched *Plectosphaerella* (noted for broad plasticity but including pathogenic strains causing agricultural damage; Raimondo and Carlucci, 2018) and *Fusicolla*. M treatment favored *Scutellinia*, while G treatment increased *Tausonia* and *Talaromyces*. These shifts indicate that CC selectively promotes or inhibits specific fungal genera, potentially disrupting or altering ecological relationships within the soil community.

### 4.3 Multivariate analysis insights and biomarker identification

PCA results confirmed the similarity between CK and M groups and the distinctiveness of D and G treatments, reinforcing the alpha and beta diversity findings. LEfSe biomarker identification provided valuable indicators: *Fusicolla*, *Fusarium*, and *Linnemannia* for D; *Tausonia*, *Talaromyces*, and *Tetracladium* for G; *Neonectria* for CK; and *Scutellinia* for M. These biomarkers are crucial for understanding the immediate effects of CC and provide a basis for long-term monitoring of soil health in relation to CC application.

### 4.4 Metabolic pathway modulation

The functional prediction of metabolic pathways revealed significant changes in response to CC treatments. The upregulation of monoacylglycerol metabolism and NAD/NADP mitochondrial interconversion, along with the downregulation of gluconeogenesis and phosphatidylglycerol biosynthesis, indicates that CC can have a profound impact on fungal metabolism. These changes in metabolic pathways may be a direct result of the interaction between CC and fungal cells or an indirect consequence of the altered soil environment caused by CC. For instance, changes in nutrient availability or pH, which can be affected by CC, may lead to the modulation of these metabolic pathways. Understanding these metabolic changes is crucial as they can ultimately influence the growth, survival, and ecological functions of soil fungi.

### 4.5 Implications for agricultural practices and future research

The findings of this study have significant implications for agricultural practices. Given that CC can have both positive and negative impacts on soil fungal communities depending on the concentration, it is essential for farmers to use CC rationally. High concentrations of CC may disrupt the soil fungal community, potentially leading to negative effects on soil health, such as reduced nutrient cycling efficiency and increased susceptibility to plant diseases. Integrated pest management strategies that combine chemical control with natural or biological control methods should be promoted to minimize the reliance on CC and maintain the stability and functionality of the soil microbial community (Zha et al., 2018).

For future research, it is necessary to further investigate the molecular and biochemical mechanisms underlying the responses of soil fungi to CC. This could involve studies on gene expression, protein - protein interactions, and the role of specific signaling pathways in fungal cells in response to CC. Long - term field experiments at multiple locations are also required to comprehensively understand the cumulative effects of CC on soil fungal diversity and ecosystem services. Additionally, more research should be conducted on the interactions between CC - induced changes in the fungal community and other components of the soil ecosystem, such as plants and bacteria, to develop a more holistic understanding of soil ecosystem functioning under CC application.

By addressing these research gaps, we can develop more sustainable agricultural practices. These practices will strive to balance the use of CC for crop protection with the maintenance of a healthy and diverse soil fungal ecosystem, ensuring long - term soil health and agricultural productivity.

## Data Availability

The datasets presented in this study can be found in online repositories. The names of the repository/repositories and accession number(s) can be found in the article/Supplementary material.
