# Supplementary material for: Impact assessment of differential chlormequat chloride exposure on soil fungal community dynamics
Source: Front Microbiol. 2025 Jul 22;16:1516835. doi: 10.3389/fmicb.2025.1516835 (PMC12321841; doi:10.3389/fmicb.2025.1516835)
Supplement: Supplementary file 1 [file Data_Sheet_1.docx]

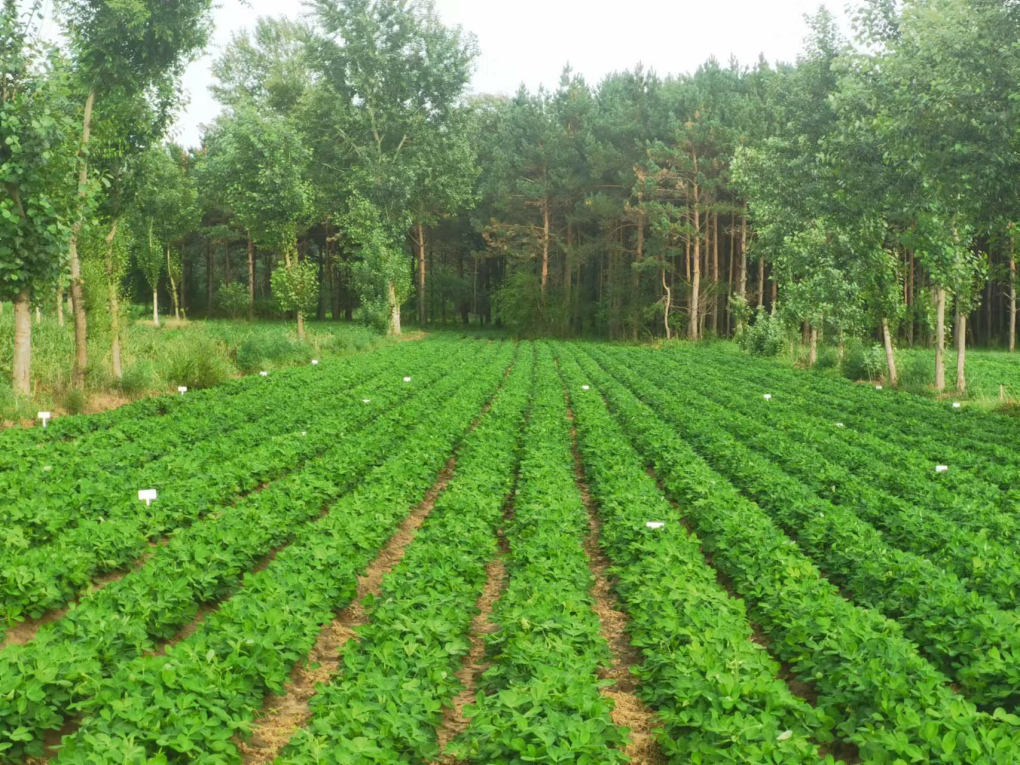


[Supplementary](https://www.frontiersin.org/journals/microbiology/articles/10.3389/fmicb.2023.1178474/full#SM1) Figure S1 Original photos of the experimental site.


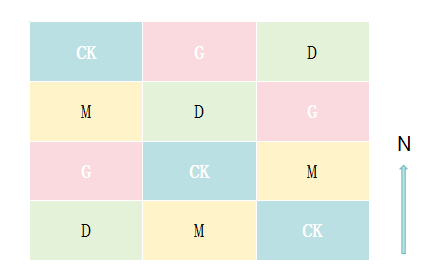
[Supplementary](https://www.frontiersin.org/journals/microbiology/articles/10.3389/fmicb.2023.1178474/full#SM1) Figure S2 Layout of Field Experimental Community.


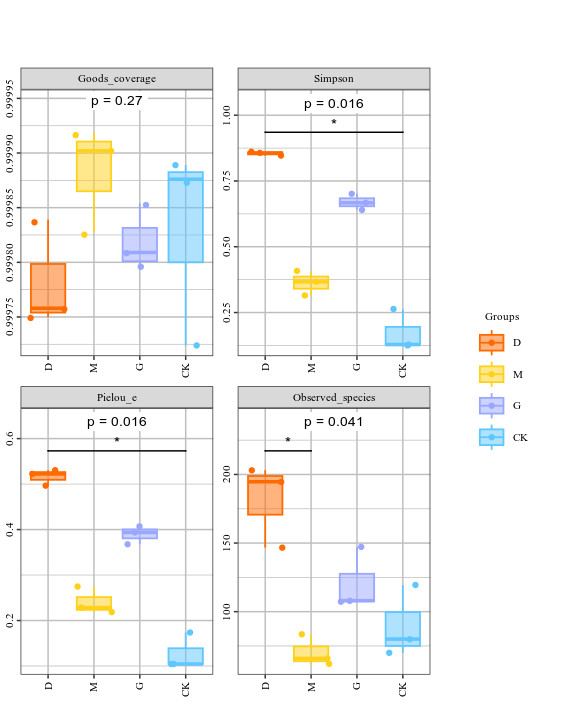


[Supplementary](https://www.frontiersin.org/journals/microbiology/articles/10.3389/fmicb.2023.1178474/full#SM1) Figure S3 Boxplots of diversity-related metrics across different concentrations of CC.
